# FROM WRINKLES TO WISE: DISRUPTING AGING PERCEPTIONS AMONG COLLEGE STUDENTS

**DOI:** 10.1093/geroni/igz038.186

**Published:** 2019-11-08

**Authors:** Katherine Bridges, Donna Fedus, Erica Michalowski

**Affiliations:** 1 AARP, Washington, District of Columbia, United States; 2 Borrow My Glasses, Inc., Madison, Connecticut, United States; 3 AARP-CT, Hartford, Connecticut, United States

## Abstract

What words come to mind when students are asked about aging? Wrinkles, grumpy, gray. What words come to mind when students are asked about aging after Disrupt Aging Classroom? Wise, wisdom, opportunity. Disrupt Aging Classroom is a successful curriculum created by AARP Connecticut and Borrow My Glasses to help dispel the myths of aging among college students in multiple disciplines. In comparison to a control group, pre-and post-test results show college students’ perceptions of aging change to a more positive frame after being exposed to this intervention administered in the classroom. There is also evidence to suggest that this program has a positive effect on behavior with students reporting they talked differently about aging and acted differently toward older adults since participating in this program. Presenters will describe the curriculum as well as the evaluation design and results collected in 2018-2019 from this successful intervention program.

